# Importance of producing economic compounds to combat cancer

**DOI:** 10.1111/1751-7915.12491

**Published:** 2017-01-26

**Authors:** Jesús Muñoz‐Rojas

**Affiliations:** ^1^ Laboratorio de Ecología Molecular MicrobianaCentro de Investigaciones en Ciencias MicrobiológicasInstituto de CienciasBenemérita Universidad Autónoma de PueblaCiudad UniversitariaEdificio 103 J, San ManuelPueblaCP72570México

## Abstract

The manuscript published by Microb Biotechnol, volume 10, highlights the relevance of the fungus *Nigrospora sphaerica*, an endophyte isolated from *Catharanthus roseus*, as an alternative source to obtain vinblastine, a compound used in chemotherapy schemes to treat several types of cancer. Authors showed that purification of vinblastine from extracts of the fungus has higher activity and yield in comparison with that obtained from the plant *Catharanthus roseus*. This work represents a biotechnological approach to obtain vinblastine with promising results to decrease the production cost.

## Highlight

One of the greatest human health problems around the world is cancer, which is defined as a collection of related diseases, where some of the body cells begin to divide without stopping and spread into surrounding tissues (National Cancer Institute, 2016). Cancer is a highly expensive disease for both individuals involved and society. The origin of this problem is multifactorial, but is associated with risks from environmental, lifestyle or behavioural exposures. Several aspects are involved in the incidence of disease including the race of individuals, familial genetic background, obesity, reproductive and hormonal history, infections caused by some viruses and bacteria, consumption of fungal polluted food, which may contain carcinogenic compounds like aflatoxins produced by fungus, consumption of alcohol, tobacco, pollution of environment by toxic compounds derived from industry, contact with herbicides and pesticides derived from agriculture, frequent consumption of food added with aromatics as conservators, for example benzoate, and others (Thun *et al*., 2014). All these factors together could mean a big problem to human health. Unfortunately, cancer incidence has been increasing and this represents a public heath for all countries (Forman and Ferlay, 2014), because an increased number of people require treatments to combat this problem. There are several treatments, but majority of them are expensive and not accessible to all people of different countries (Gelband *et al*., 2016). One treatment frequently used as the first election is chemotherapy, and chemicals used should be cheaper to increase the population attended by this treatment. Chemotherapy uses several compounds depending on the kind of cancer, severity and choice of patient.

**Figure 1:**
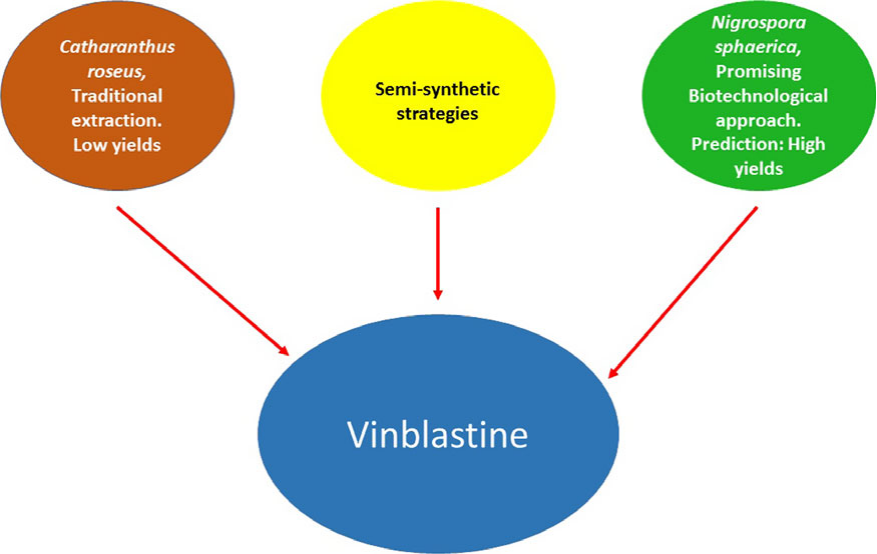
Different ways to obtain vinblastine.

Vinblastine is an alkaloid marketed as anticarcinogen compound frequently used in ABVD chemotherapy to treat several kinds of cancer including the Hodgkin's lymphoma (Gunther *et al*., 2016). This compound was traditionally extracted from *Catharanthus roseus* (Miura *et al*., 1988), but with low yields. The semi‐synthetic strategies are more efficient methods to obtain vinblastine (Kuehne *et al*., 1991; Verma *et al*., 2007), but these require a very expensive technology. On the other hand, microbial strains that produce compounds of high value also represent an excellent strategy for biotechnological large‐scale production. Therefore, in this number of Microbial Biotechnology, an alternative strategy to produce vinblastine from *Nigrospora sphaerica*, an endophyte of *C. roseus*, is showed (Wahida *et al*. 2016) (Fig. 1). Authors carried out the purification of vinblastine from a crude mycelia extract and tested for cytotoxicity activity using MTT assays with breast cell line cancer (MDA‐MB 231). The purified compound from crude mycelia extract of *N. sphaerica* had higher cytotoxicity activity than traditional extract obtained from *C. roseus*. The production of vinblastine by the fungus is superior to that obtained from plants, and this strategy could reduce production cost and concomitantly reduce cancer treatments, which is crucial while the most appropriate treatments are sought. In addition, this biotechnological strategy also represents an ecological process that prevents destruction of plants and does not put them in danger of extinction.

Perhaps chemotherapy is not the best treatment for cancer; in fact new treatments such as the use of superconducting cyclotron for proton therapy have been more effective (Knapp and Dash, 2016; Ren *et al*., 2016). However, it is important to note that these kinds of treatments are still under research and they are not available for the majority of the population yet. While this occurs, the production of vinblastine through the fungus *N. sphaerica* is a very important step forward for biotechnology and global health.

## Conflict of Interest

None declared.
